# Identification of SDC1 as a Key Regulator and Therapeutic Target in Rheumatoid Arthritis via JAK2‐STAT3 Pathway

**DOI:** 10.1111/1756-185x.70524

**Published:** 2026-01-29

**Authors:** Gan Cao, Zhihui Wu, Yatao Du, Dongxue Dai, Yang Sun, Xi Jia, Huixin Cai

**Affiliations:** ^1^ Medical Laboratory Department Baoding No. 1 Central Hospital Baoding City Hebei Province China; ^2^ The Pathology Department Xi'an Chest Hospital Xi'an City Shanxi Province China

**Keywords:** collagen‐induced arthritis, rheumatoid arthritis, Syndecan‐1

## Abstract

**Introduction:**

Rheumatoid arthritis (RA) is a chronic autoimmune disorder with unclear molecular mechanisms, complicating early diagnosis and treatment. This study aimed to identify hub genes and pathways driving RA pathogenesis and assess their therapeutic potential.

**Methods:**

Gene expression datasets related to RA were retrieved from the Gene Expression Omnibus (GEO) database. Differentially expressed genes (DEGs) were identified and analyzed by functional enrichment and protein–protein interaction network construction. Machine learning approaches, including LASSO regression, random forest, and SVM‐RFE, were used to screen hub genes. Pathway associations were explored using Gene Set Enrichment Analysis (GSEA). Experimental validation was performed in collagen‐induced arthritis (CIA) rat models and MH7A synovial fibroblast cells through Western blot and functional assays.

**Results:**

A total of 106 DEGs were identified in RA synovial tissues, including 76 upregulated and 30 downregulated genes. Enrichment analyses revealed involvement in cytokine–cytokine receptor interaction, lymphocyte‐mediated immunity, and immunoglobulin complexes. SDC1 emerged as a key hub gene across all three machine learning methods. GSEA indicated its significant correlation with the JAK–STAT pathway. In CIA rats, SDC1 expression was markedly elevated alongside p‐JAK2 and p‐STAT3 levels. Silencing SDC1 in MH7A cells reduced cell proliferation, decreased p‐JAK2 and p‐STAT3 expression, and promoted apoptosis.

**Conclusions:**

This study identifies SDC1 as a central hub gene in RA pathogenesis through activation of the JAK2–STAT3 signaling pathway. These findings highlight SDC1 as a potential biomarker for early diagnosis and a promising target for therapeutic intervention, providing new insights into RA management.

## Keypoints

- SDC1 identified as a key hub gene in RA by integrated bioinformatics and machine learning.
- SDC1 promotes RA progression via activation of the JAK2–STAT3 pathway.
- Targeting SDC1 may provide a novel diagnostic and therapeutic strategy for RA.

## 1 Background

Rheumatoid arthritis (RA) is a chronic, systemic autoimmune disorder [1] that is primarily characterized by inflammatory responses and structural damage affecting the synovial membranes within the joints [2], which may ultimately result in joint deformities and loss of function [3]. Immune abnormalities, genetic factors, and environment are believed to play important roles in pathogenesis [4]. As the early symptoms of RA are not obvious, early diagnosis mainly depends on clinical manifestations [5], laboratory tests (such as rheumatoid factor), and imaging examinations [6]. However, these methods have certain limitations in the early stage of the disease [7]. Therefore, identifying new molecular markers to improve the accuracy of early diagnosis of RA and identifying new molecular targets are vital for the timely detection and management of RA.

In recent years, bioinformatic technology has played a more significant role in disease research [8] and drug development [9]. Through the comprehensive analysis of high‐throughput sequencing and publicly available gene expression databases, disease‐related differentially expressed genes (DEGs) can be systematically screened [10], and machine learning can be used to further mine key genes. Although certain progress has been made in RA research, there remain some limitations, including the lack of multi‐omics integration, imperfect key gene screening strategies, and insufficient experimental validation of bioinformatics predictions. To address these gaps, our study combined multiple GEO datasets and advanced machine learning methods (LASSO regression, SVM‐RFE, and random forest) to systematically identify key genes and signaling pathways. Importantly, the predicted genes were further validated in both cell (MH7A) and animal (CIA rat) models, providing robust functional evidence. This integrated approach enhances the stability, credibility, and translational relevance of our findings, representing a significant advance over previous studies.

## 2 Materials and Methods

### 2.1 Data Normalization and Identification of Differentially Expressed Genes

Data were sourced from the GEO database, specifically from the datasets GSE77298 and GSE55235 (both designated as training datasets) as well as GSE1919 and GSE12021 (identified as validation datasets). The microarray data corresponding to GSE77298 and GSE55235 underwent processing, normalization, and batch correction using the “sva” package within R software. Subsequently, we employed the “limma” package for the identification of differential genes, with statistically significant genes defined by an adjusted *p* < 0.05 and a log2 fold change greater than 2. Visualization of differentially expressed genes was achieved through the “ggplot2” package and “pheatmap.”

### 2.2 Analysis of Functional Enrichment and Construction of PPI Networks

To investigate the functions and pathways associated with DEGs, the “clusterProfiler” R software package was used to conduct enrichment assessments of the Gene Ontology (GO) and Kyoto Encyclopedia of Genes and Genome (KEGG) pathways. Next, the STRING database was used to visualize the PPI network of DEGs, and the resulting network was displayed using Cytoscape software (version 3.9). Using Cytoscape software, an in‐depth analysis of the PPI network generated by the STRING website was performed using the CytoNCA plugin to identify key genes, known as HubGenes.

### 2.3 Identification and Verification of Core Genes

Three machine learning methods—Random Forest, LASSO regression, and SVM‐RFE—were employed to precisely identify important hub genes in RA. For LASSO regression, the regularization parameter *λ* was determined via 10‐fold cross‐validation to minimize the mean cross‐validated error. In SVM‐RFE, a linear kernel function was used, and recursive feature elimination was performed iteratively to rank genes based on their contribution to classification accuracy. For Random Forest, 500 trees were constructed, and the mean decrease in Gini index was used to evaluate feature importance. The results obtained from these three methods were compared using the Venn diagram tool, and the overlap among the three methods suggested potential hub genes for diagnosing RA. The external datasets GSE1919 and GSE12021 were utilized to confirm the hub genes.

### 2.4 Analysis of Gene Set Enrichment Focusing on a Single Gene

The Spearman correlation method was used to determine the association between core genes and other genes throughout the entire genome, and the genes were ranked according to the correlation coefficients to generate an ordered gene list. Subsequently, GSEA was conducted using R packages, including clusterProfiler, with the c2.cp.kegg_medicus.v2024.1. Hs.symbols.gmt dataset from the MSigDB database serving as the reference gene set, and significantly enriched pathways were obtained. Visual analysis was performed through the creation of enrichment plots to uncover the potential biological roles and associated signaling pathways of the core genes.

### 2.5 Cell Culture and siRNA Transfection

The fibroblast‐like synoviocyte cell line MH7A was purchased from Meisen Cell Biological Technology (Hangzhou, China). MH7A cells were cultured in 1640 medium (containing 10% FBS). siSDC1 was transferred to cells for 48 h using Lipofectamine 2000 according to the instructions.

### 2.6 RT‐qPCR

Total RNA was extracted using TRIzol, followed by reverse transcription into cDNA according to the manufacturer's protocol. Quantitative PCR was conducted using the qPCR detection system. The primer sequences used in this study were as follows:

*Homo sapiens* SDC1 Forward primer (5′ to 3′): CCACCATGAGACCTCAACCC.

Reverse primer (5′ to 3′): GCCACTACAGCCGTATTCTCC.

*Homo sapiens* *β*‐actin Forward primer (5′ to 3′): CCTGGCACCCAGCACAAT.

Reverse primer (5′ to 3′): GGGCCGGACTCGTCATAC.

### 2.7 Western Blotting Analysis

Total proteins were extracted from cells or tissues, separated by SDS‐PAGE, transferred to PVDF membranes, blocked, and incubated with primary and secondary antibodies. The proteins were visualized using an ECL chemiluminescence detection system.

### 2.8 Apoptosis Experiment

The cells that were transfected with siRNA were collected. Apoptosis was assessed using an apoptosis detection kit. 1X Binding Buffer was prepared following the protocol provided in the kit, and the cell concentration was set to 3 × 10^6^/ml. Annexin V‐FITC was added and the cells were incubated for 15 min. Once the incubation period was complete, cells were washed with 1X Binding Buffer. Subsequently, 7‐AAD Viability Staining Solution was added, and analysis was conducted using flow cytometry.

### 2.9 Edu Analysis

After transfecting siRNA into MH7A cells for 48 h, the EdU working solution was added and incubated for 2 h to incorporate it into the DNA of proliferating cells. After incubation, the medium was removed, and the cells were fixed for 15 min and washed three times. 0.3% Triton X‐100 was added, incubated for 15 min, the cells were washed three times, the Click reaction system was used to label EdU, and the cells were incubated for 30 min. Flow cytometry was used to detect the EdU‐488 fluorescence signal, allowing the assessment of MH7A cell proliferation.

### 2.10 Construction of CIA Rat Model

A total of 16 male Sprague–Dawley (SD) rats (6–8 weeks old, 180–200 g) were purchased from Beijing Huafukang Biotechnology Co. Ltd. and adaptively raised for 1 week under standard laboratory conditions (22°C ± 2°C, 12 h light/dark cycle, free access to food and water). To construct the collagen‐induced arthritis (CIA) model, a mixture of bovine type II collagen and complete Freund's adjuvant (CFA) was prepared and injected subcutaneously at the base of the tail and the left sole for the primary immunization (day 0). A booster immunization was administered on day 7 into the right paw. Rats were observed every 3 days for clinical symptoms, including joint redness, swelling, stiffness, and limited movement, and the degree of joint swelling was measured regularly using a standardized scoring system. Arthritis severity was assessed every 3 days after the booster immunization according to a standard arthritis index (AI) scoring system ranging from 0 to 4 for each paw (0 = normal, 1 = mild swelling and erythema, 2 = moderate swelling, 3 = pronounced swelling, 4 = severe swelling and deformity), with a maximum total score of 16 per animal.

### 2.11 HE Staining

A month after the first immunization, synovial tissue was extracted from the joints and preserved in a 4% paraformaldehyde solution for 24 h. This was followed by embedding in paraffin and cutting into sections. Hematoxylin–eosin (HE) staining was conducted following the manufacturer's protocol, and pathological changes in synovial tissue were examined under an optical microscope to assess the characteristics associated with arthritis pathology.

### 2.12 Molecular Docking

Molecular docking was used to analyze the interactions between the key proteins and core targets in the relevant pathways. The three‐dimensional structures of the proteins were obtained from the Protein Data Bank, and docking analysis was performed using AutoDock to predict the interaction patterns between the core targets and key pathway proteins, which were visualized using PyMOL software. The docking results clearly showed the amino acid residues involved in the binding between SDC1 and JAK2, including ER‐171, SER‐919 and GLU‐845 et al., Strong hydrogen bonds and hydrophobic interactions were observed between SDC1 and JAK2, indicating a stable and specific binding mode.

### 2.13 Statistical Analysis

Quantitative data are presented as mean ± standard deviation (SD). Statistical analyses were conducted using R software (version 4.0) and GraphPad Prism 8, with *p* < 0.05 regarded as indicative of statistical significance.

## 3 Results

### 3.1 Identification of DEGs Between RA and Normal Controls

**FIGURE 1:**
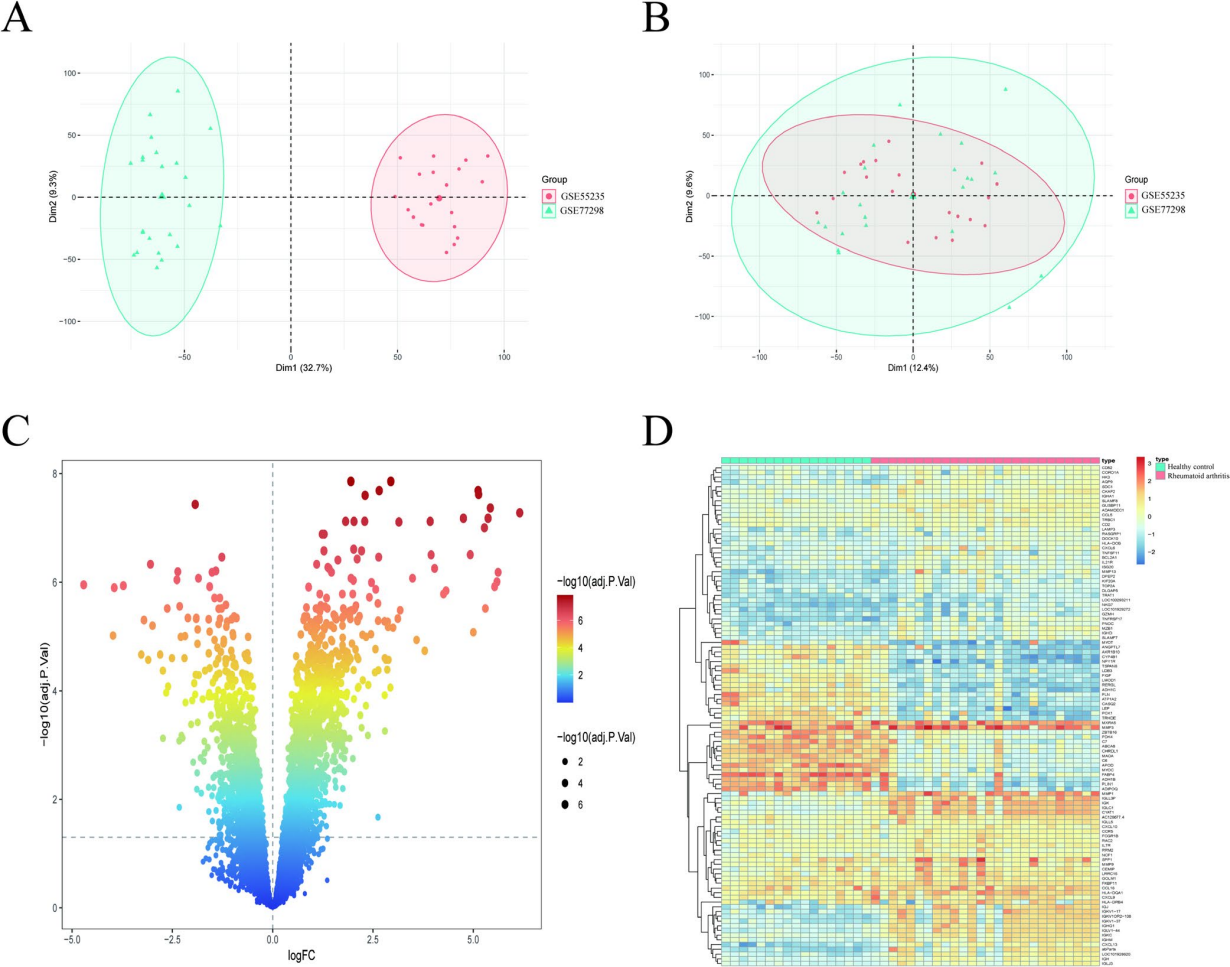
Merging and DEG analysis of GSE datasets. (A) The principal component analysis (PCA) results show the distribution of all samples before normalization. (B) The PCA plot shows the sample distribution after normalization. (C) The volcano plot represents all the normalized mRNA expression data. (D) The heatmap shows the DEGs in the synovial tissue of RA patients.

We merged datasets GSE55235 and GSE77298 and adjusted for batch effects to obtain a normalized combined matrix. There were some differences in the sample distribution between these two datasets (Figure 1A), which may hinder subsequent analysis. The merged dataset after “sva” package processing showed strong homogeneity, indicating high consistency and uniformity (Figure 1B). Based on the filtering criteria of adj. *p* < 0.05 and |log2FC| > 2, a total of 76 genes were upregulated while 30 genes were downregulated when comparing RA to normal controls Table S1. The DEGs between RA and normal controls were visually illustrated using a volcano plot (Figure 1C) and heatmap (Figure 1D).

### 3.2 Enrichment Analysis of DEGs

**FIGURE 2:**
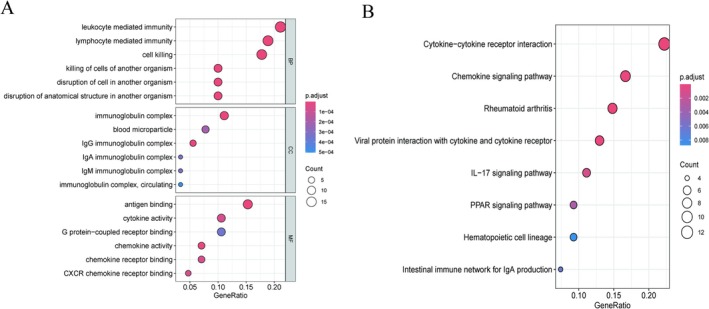
Functional enrichment analysis of differential genes between RA and normal controls. (A) The results of GO pathway enrichment analysis for the differential genes. (B) The results of KEGG pathway enrichment analysis for the differential genes.

The GO enrichment analysis results (Figure 2A) show that the DEGs identified between the RA and normal control groups were predominantly associated with pathways including leukocyte‐mediated immunity, lymphocyte‐mediated immunity, and immunoglobulin complexes, while the KEGG enrichment results (Figure 2B) indicated that the DEGs were primarily enriched in rheumatoid arthritis and chemokine signaling pathway, etc.

### 3.3 Identifying Potential Diagnostic Biomarkers for RA Through Machine Learning

**FIGURE 3:**
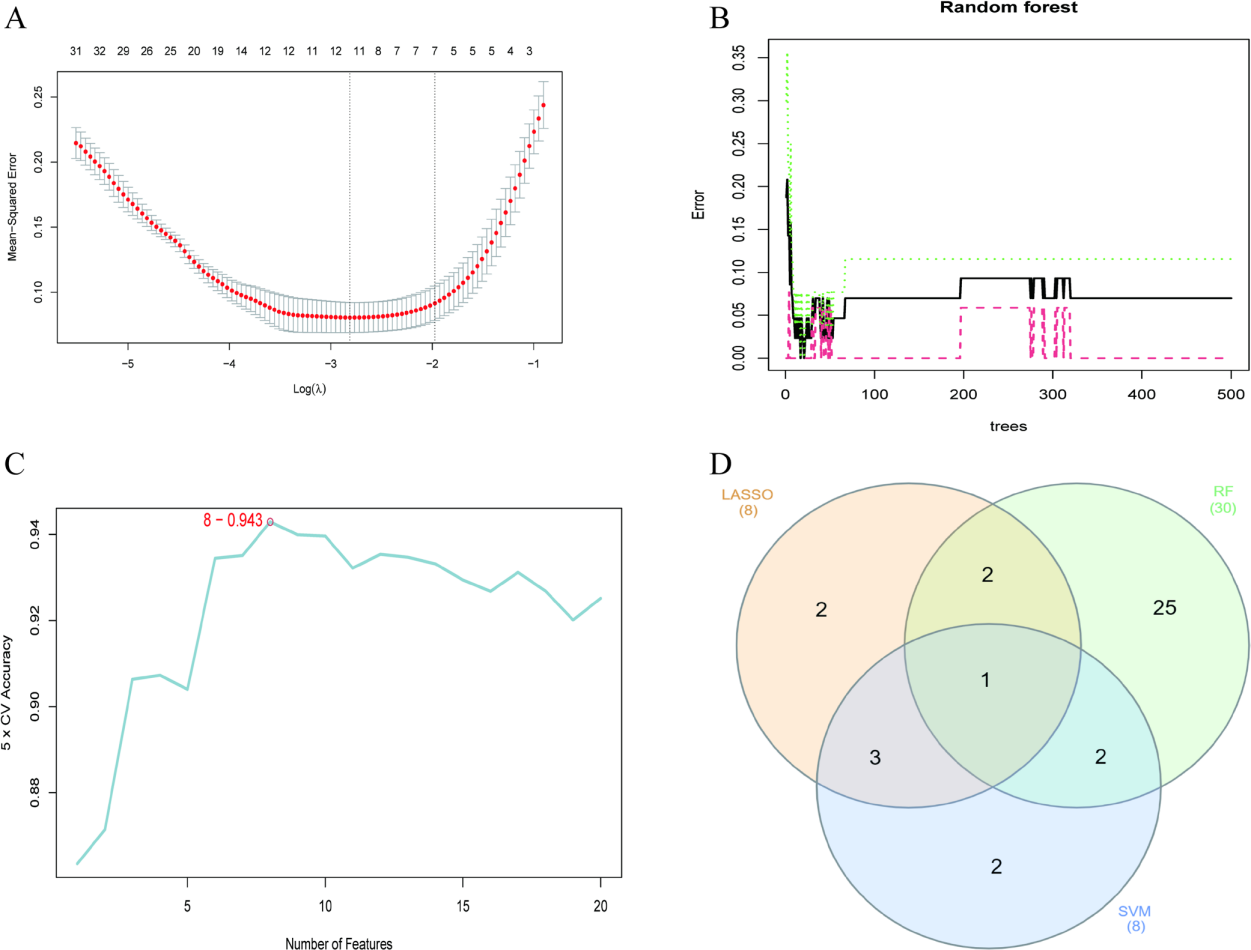
Identifying key genes using machine learning techniques: (A) LASSO regression analysis. (B) RF algorithm application. (C) Feature selection via the SVM‐RFE method. (D) Venn diagram illustrating the shared feature genes identified by SVM‐RFE, Lasso, and RF methods.

Lasso regression was applied to feature gene selection. The plot displays binomial deviance against Log(λ), with the optimal λ identified at the point of minimum deviance (Figure 3A). We then used a random forest algorithm to identify the key genes (Figure 3B). Finally, we employed the SVM‐RFE technique (Figure 3C) to identify the essential genes distinguishing RA from normal controls. Venn diagram showing the intersection of core genes of the three different machine learning methods (SVM‐RFE, Lasso, and RF). A single characteristic gene, SDC1, was found to be common across all methods (Figure 3D).

### 3.4 Construction of PPI Network

PPI analysis was conducted using the STRING database to explore the interactions among all identified DEGs, and a PPI network comprising 262 edges and 75 nodes was generated and visualized using Cytoscape software Figure S1A. The CytoNCA algorithm was used to predict the key DEGs. Finally, 22 DEGs were identified as key genes Figure S1B.

### 3.5 Validation of the Hub Gene SDC1 Using External Datasets

To confirm the reliability and significance of the results, two external datasets (GSE12021 and GSE1919) were used to validate the hub genes. SDC1 was significantly overexpressed in RA synovial tissue (Figure S2).

### 3.6 Gene Set Enrichment Analysis

**FIGURE 4:**
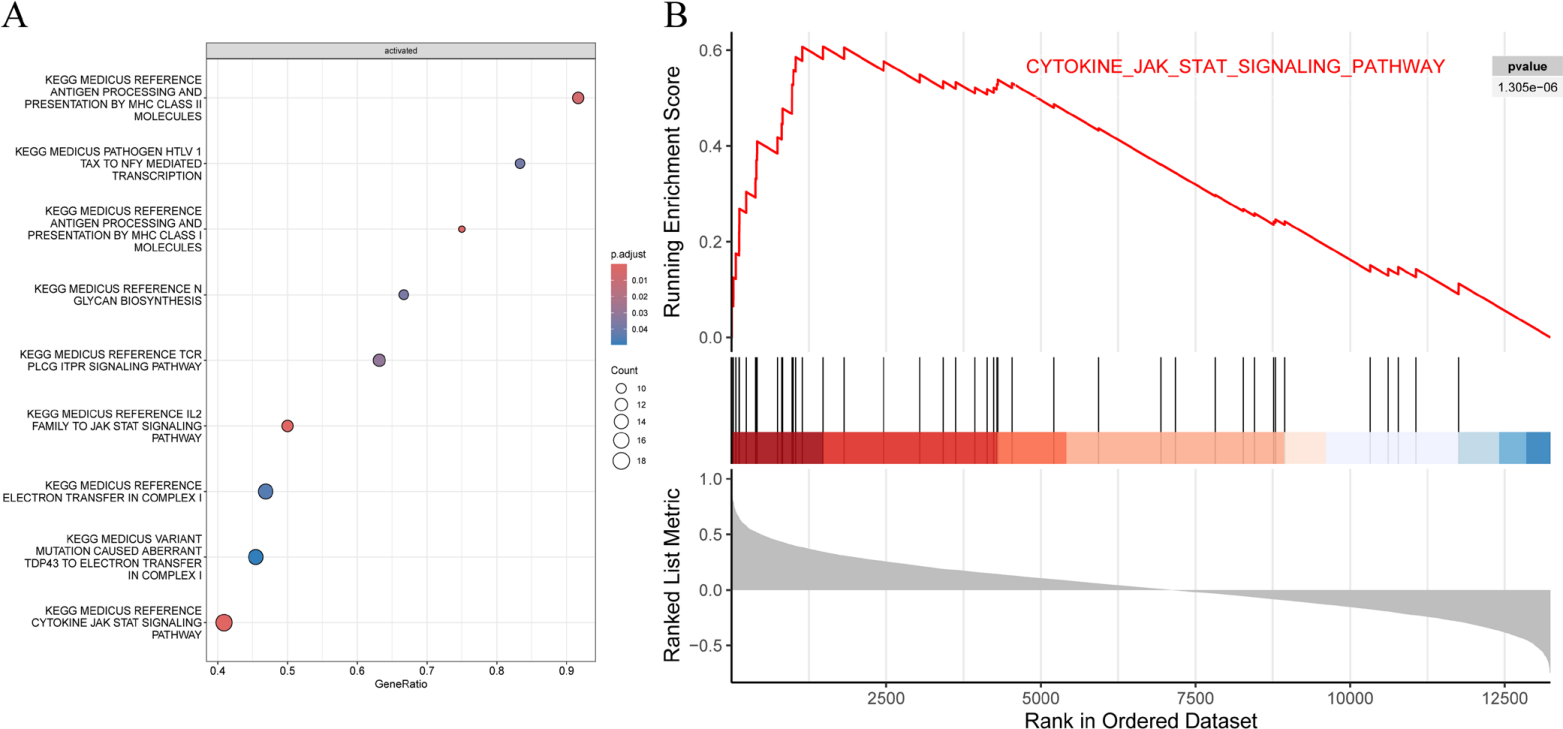
Gene set enrichment analysis. (A) Kyoto Encyclopedia of Genes and Genomes (KEGG) enrichment analysis of SDC1. (B) SDC1 was significantly enriched in CYTOKINE_JAK_STAT_SIGNALING_PATHWAY.

Spearman's correlation analysis was used to calculate the correlation between SDC1 and other genes, and GSEA functional enrichment analysis was performed. The results from GSEA (Figure 4A,B) indicated a significant enrichment of SDC1 in eight KEGG signaling pathways, notably within the CYTOKINE_JAK_STAT_SIGNALING_PATHWAY. Consequently, we hypothesized that SDC1 might be involved in the pathogenesis of RA through the CYTOKINE_JAK_STAT_SIGNALING_PATHWAY.

### 3.7 SDC1 Promotes Arthritis in CIA Rats via the JAK2‐STAT3 Signaling Pathway

**FIGURE 5:**
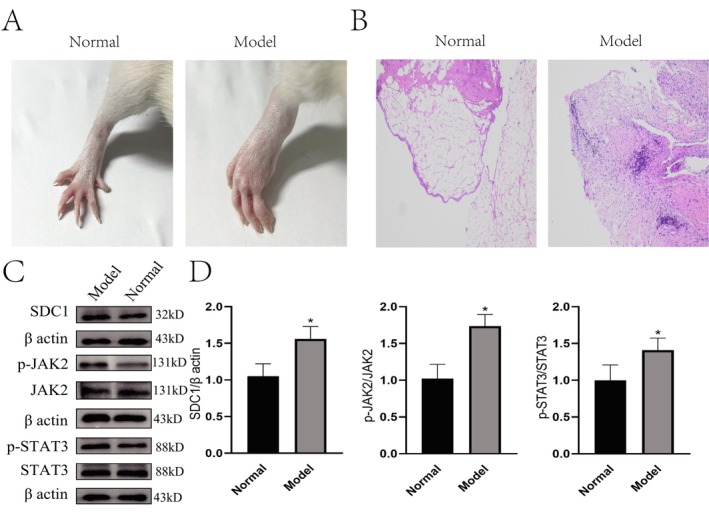
SDC1 and JAK2‐STAT3 pathways were significantly activated in CIA rats. (A) Representative images of the hind paw. (B) Pathological images stained with hematoxylin and eosin (H&E) showed significant synovial hyperplasia in the joints of the CIA model group. (C) Representative Western blotting bands of SDC1, pJAK2, pSTAT3, JAK2, STAT3 proteins. (D) Quantitative analysis of SDC1, pJAK2, and pSTAT3 proteins.

Janus kinase (JAK) consists of four members: JAK1, JAK2, JAK3, and TYK2. Consequently, we conducted molecular docking studies of SDC1 with each of the four JAK family members using AutoDock software. The results of the molecular docking analysis (Figure S3 and Table S2) indicated that SDC1 exhibited the strongest binding affinity with JAK2. We also established a rat model of CIA. After two immunizations, the joints of the CIA rats were significantly swollen (Figure 5A). The HE staining results (Figure 5B) showed synovial hyperplasia and a large amount of inflammatory cell infiltration in the model group, indicating that the model was successfully constructed. The expression of SDC1, pJAK2, pSTAT3, JAK2, and STAT3 was detected by immunoblotting (Figure 5C,D). The results from the WB analysis indicated that in comparison to the normal group, the levels of SDC1, pJAK2, and pSTAT3 in the synovial membrane of joints were markedly elevated in the CIA model group.

### 3.8 The Effect of SDC1 Silencing on MH7A Cells

**FIGURE 6:**
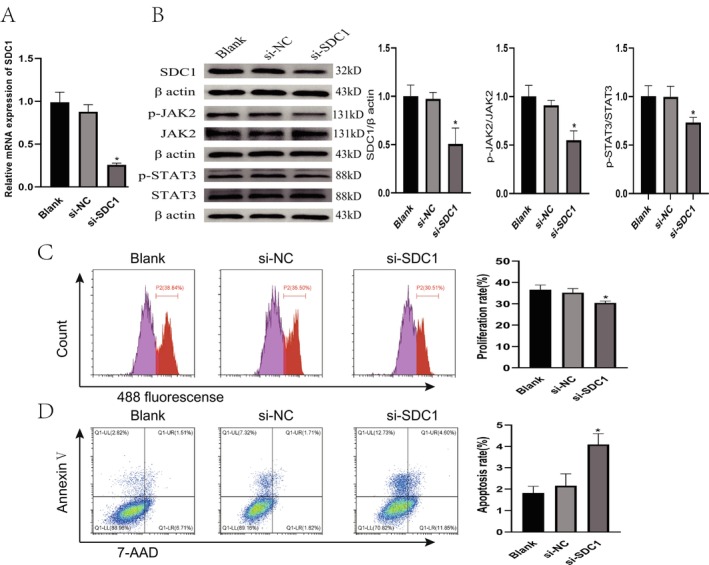
The effect of SDC1 silencing on JAK2‐STAT3 signaling pathway, proliferation, and apoptosis in MH7A cells. (A) Validation of si‐SDC silencing efficiency. (B) Relative expression of SDC1, pJAK2, pSTAT3, JAK2, and STAT3 proteins after si‐SDC1 transfection. (C) MH7A cell proliferation was detected by Edu method after si‐SDC1 transfection. (D) MH7A cell apoptosis ratio was detected by flow cytometry after si‐SDC1 transfection.

To further verify that SDC1 may be involved in the pathogenesis of RA through the modulation of the JAK2‐STAT3 signaling pathway, we used small interfering RNA (si‐RNA) technology to interfere with the expression of SDC1 and observed its effects on the expression of pJAK2 and pSTAT3 as well as cell phenotype. Before the experiment, we verified the transfection efficiency of si‐RNA. Our results showed that the levels of SDC1 mRNA (Figure 6A) and protein (Figure 6B) significantly decreased after transfection with si‐SDC1. Immunoblotting results revealed that the levels of pJAK2 and pSTAT3 in MH7A cells were significantly reduced after transfection with si‐SDC1 (Figure 6B). Flow cytometry results demonstrated that transfection of MH7A cells with si‐SDC1 significantly reduced their proliferation (Figure 6C) and enhanced their apoptosis (Figure 6D). These results suggest that SDC1 may affect abnormal proliferation of fibroblasts through the JAK2‐STAT3 pathway.

## 4 Discussion

Rheumatoid arthritis (RA) is a chronic autoimmune disease that results from the combined effects of genetic susceptibility, environmental exposures, and aberrant immune responses [11]. Despite advances in diagnosis and therapy, early detection and targeted treatment remain significant challenges [12]. This study combined bioinformatic analysis with experimental validation and identified SDC1 as a key gene involved in the pathogenesis of RA, indicating that it may play a role in disease progression through the JAK2‐STAT3 signaling pathway.

### 4.1 Syndecan 1 (SDC1)

[13] is widely present on the surface of different types of cells [14] and is crucial for biological processes such as cell signaling [15], cell adhesion [16], proliferation [17] and regulation of immune responses [18]. Research indicates that SDC1 serves a significant regulatory function in autoimmune disorders [19], mainly involving immune cell function [20], inflammatory response [21] and tissue damage. Recent studies highlight the role of Syndecan‐1 (SDC‐1) in rheumatoid arthritis (RA) pathogenesis [22]. Shahrara et al. demonstrated that the syntenin‐1/SDC‐1 axis modulates RA progression by regulating the crosstalk between glycolytic macrophages and Th1 cells, linking metabolic reprogramming to inflammatory responses [23]. In contrast, Abdo Jurjus et al. report that SDC‐1 deficiency exacerbates experimental RA, with knockout models showing increased TNF‐*α* expression, severe joint erosion, and reduced mast cell activity [24]. These seemingly contradictory findings suggest that SDC‐1 may be influenced by the cellular microenvironment, which may serve as both a mediator of inflammatory pathways and a protective factor in joint homeostasis. In SLE, SDC1 regulates B cell activation and participates in immune complex deposition, which is closely linked to the development of SLE [25]. Research indicates that serum SDC‐1 levels are elevated in SLE patients experiencing nephritis, suggesting that SDC‐1 could serve as a valuable serum biomarker for active lupus nephritis (LN) [26]. The pleiotropic role of SDC1 extends across multiple autoimmune disorders [27]. In inflammatory bowel diseases (IBD), SDC1 maintains the integrity of the intestinal barrier and regulates inflammation, and changes in its expression can impair epithelial repair and immune cell recruitment [28]. Similarly, in multiple sclerosis (MS), SDC1 regulates T cell activation and myelin damage, influencing central nervous system inflammation [29]. Patients with Sjögren syndrome exhibit abnormal SDC1 expression in salivary glands, where they disrupt epithelial function and promote immune cell infiltration, exacerbating tissue damage [30]. These findings collectively suggest that SDC1 is a central regulator of autoimmune pathogenesis across diverse tissues. Our research identified 106 genes that were differentially expressed in RA through an analysis of differentially expressed genes (DEG). SDC1 was identified as a key gene in RA using three machine learning methods: SVM‐RFE, LASSO, and random forest. This robust computational prediction strongly implicates SDC1 in the disease's pathological mechanisms, suggesting it may serve as a critical regulator of RA progression. In summary, the role of SDC1 appears to be context‐dependent across autoimmune diseases. In systemic lupus erythematosus (SLE), SDC1 has been implicated in modulating B cell differentiation and autoantibody production, while in inflammatory bowel disease (IBD), it participates in epithelial barrier maintenance and regulation of mucosal immune responses. These observations suggest that while SDC1 is broadly involved in immune regulation, the downstream signaling pathways and cellular targets differ by disease context, highlighting a degree of pathway specificity in RA.

Taken together, these insights emphasize the complex and multifaceted roles of SDC1, supporting its potential as a therapeutic target in RA. Targeting SDC1 may need to consider both its protective and pathogenic aspects to achieve precise modulation of synovial inflammation without disrupting homeostatic functions.

Gene set enrichment analysis (GSEA) indicated a strong association between SDC1 and the JAK–STAT signaling pathway. The Janus kinase‐signal transducer and activator of transcription (JAK–STAT) pathway plays a crucial role in the transduction of cytokine‐mediated signals [31] and is extensively involved in various physiological processes [32]. This pathway is composed of cytokine receptors, JAK kinases, and STAT transcription factors (STAT1‐STAT6) [33]. The signal transduction mechanism mainly includes the binding of cytokines to their receptors, thereby regulating the transcription of target genes [34]. Dysregulated activation of the JAK–STAT signaling pathway has been associated with the pathogenesis of numerous disorders, particularly autoimmune diseases [35], cancer [36], etc. [37]. Several studies have shown that the JAK–STAT signaling pathway plays a central role in RA pathogenesis, where it regulates inflammatory responses, drives immune cell activation, and facilitates synovial cell proliferation. Persistent activation of this pathway during the pathogenesis of RA contributes to the progression of joint damage through elevated matrix metalloproteinase gene expression, chondrocyte apoptosis, and resistance of synovial tissue to apoptosis [38]. Targeted inhibition of the JAK–STAT pathway ameliorates clinical symptoms and slows disease progression in rheumatoid arthritis [39].

Molecular docking analysis revealed that SDC1 exhibited the strongest binding affinity for JAK2. Western blot analysis revealed a significant upregulation of SDC1, p‐JAK2, and p‐STAT3 in the synovial tissue of rats in the model group, suggesting that SDC1 may contribute to RA pathogenesis by activating the JAK2‐STAT3 signaling pathway, thereby promoting synovial cell proliferation and inflammatory responses.

To further investigate the role of SDC1, we reduced SDC1 expression in the MH7A cell line. Our experimental findings revealed that the knockdown of SDC1 notably decreased cell proliferation, lowered the levels of p‐JAK2 and p‐STAT3, and induced apoptosis in the cells. These results provide additional evidence that SDC1 is critically involved in the pathogenesis of RA through modulation of the JAK2‐STAT3 signaling pathway. Therefore, SDC1 may serve as a promising therapeutic target for the management of RA. By inhibiting SDC1 or its related signaling pathways, it may be feasible to effectively curb the abnormal growth and inflammatory response of synovial cells, thereby mitigating the progression of RA.

In addition to its upstream regulatory role in the JAK2–STAT3 signaling pathway, SDC1 has been reported to influence several downstream effectors implicated in RA pathogenesis. Previous studies have shown that SDC1 can modulate the expression of matrix metalloproteinases (MMP‐1, MMP‐3, MMP‐9), contributing to extracellular matrix degradation and cartilage destruction. Moreover, SDC1 has been associated with regulation of pro‐inflammatory cytokines, including IL‐6, TNF‐α, and IL‐1*β*, thereby promoting synovial inflammation and fibroblast activation. These observations suggest that SDC1 may act as a central node linking JAK2–STAT3 signaling to multiple pathogenic effectors in RA, providing a mechanistic framework for understanding its potential as a therapeutic target.

This study underscores the pivotal role of SDC1 in the pathogenesis of rheumatoid arthritis (RA), supported by bioinformatics analysis and experimental validation. It identified how SDC1 regulates the progression of RA through the JAK2‐STAT3 signaling pathway.

However, this study had several limitations remain. Although this study confirmed the role of SDC1 in CIA rat models and MH7A cells, its role in rheumatoid arthritis patients still needs further investigation through large‐scale clinical studies. The precise regulatory mechanisms of SDC1 in RA are not yet fully understood, and future research should focus on exploring its upstream regulators and downstream effectors.

This study combined bioinformatics, machine learning, and experimental validation to explore the role of SDC1 in rheumatoid arthritis (RA). This suggests that SDC1 may regulate the pathological process of RA through the JAK–STAT signaling pathway. This discovery not only identifies new potential targets for diagnosing and treating RA but also creates new pathways for exploring the function of SDC1 in autoimmune disorders.

## 5 Conclusions

This study identified SDC1 as a key gene in the pathogenesis of rheumatoid arthritis. By integrating bioinformatics analysis with experimental validation, we demonstrated that SDC1 promotes disease progression via the JAK2‐STAT3 signaling pathway. These findings suggest that SDC1 may serve as a potential biomarker for early diagnosis and a novel target for therapeutic intervention in rheumatoid arthritis.

## Author Contributions

**Gan Cao:** visualization, writing original draft. **Zhihui Wu:** data curation, formal analysis. **Yatao Du:** formal analysis. **Dongxue Dai:** methodology. **Yang Sun:** software. **Xi Jia:** software. **Huixin Cai:** writing, review and editing, conceptualization.

## Funding

This study was supported by the Natural Science Foundation of Hebei (H2021104017).

## Disclosure

Animal Research: All animal experiments were approved by the Animal Ethics Committee of Ningxia Medical University (approval number: IACUC‐NYLAC‐2024‐008). All procedures were performed in accordance with the Regulations for the Care and Use of Laboratory Animals and the ARRIVE guidelines. Every effort was made to minimize animal suffering and to reduce the number of animals used.

## Ethics Statement

The Medical Ethics Committee of Baoding No. 1 Central Hospital reviewed and approved the study protocol (approval no. [2021]008). All participants provided informed consent at the time of enrollment.

## Conflicts of Interest

The authors declare no conflicts of interest.

## Supporting information


**Figure S1:** Construction of protein–protein interaction network and identification of key genes. (a) The PPI network of differentially expressed genes. (b) The prediction results of CytoNCA algorithm, CXCL13, CCL5 and CXCL10 identified as core hub genes.

**Figure S2:** Validation of Hub Genes SDC1. Comparison of SDC1 expression in normal and RA synovial tissues using two external datasets (GSE12021 and GSE1919).

**Figure S3:** The molecular docking results of SDC1 and JAK2. Predicted SDC1–JAK2 docking model highlighting key interface residues (SER‐171, SER‐919, GLU‐845 et al.) and the main stabilizing forces—hydrogen bonds and hydrophobic interactions.

**Table S1:** Complete list of differentially expressed genes (DEGs) identified in the study.

**Table S2:** Binding Energy of SDC1 and JAK family.

## Data Availability

The data that support the findings of this study are available from the corresponding author upon reasonable request.
