# Supplementary material for: Identification of SDC1 as a Key Regulator and Therapeutic Target in Rheumatoid Arthritis via JAK2‐STAT3 Pathway
Source: Int J Rheum Dis. 2026 Jan 29;29(2):e70524. doi: 10.1111/1756-185x.70524 (PMC12853146; doi:10.1111/1756-185x.70524)
Supplement: Supplementary file 1 — Figure S1: Construction of protein–protein interaction network and identification of key genes. (a) The PPI network of differentially expressed genes. (b) The prediction results of CytoNCA algorithm, CXCL13, CCL5 and CXCL10 identified as core hub genes. [file APL-29-e70524-s004.pdf]

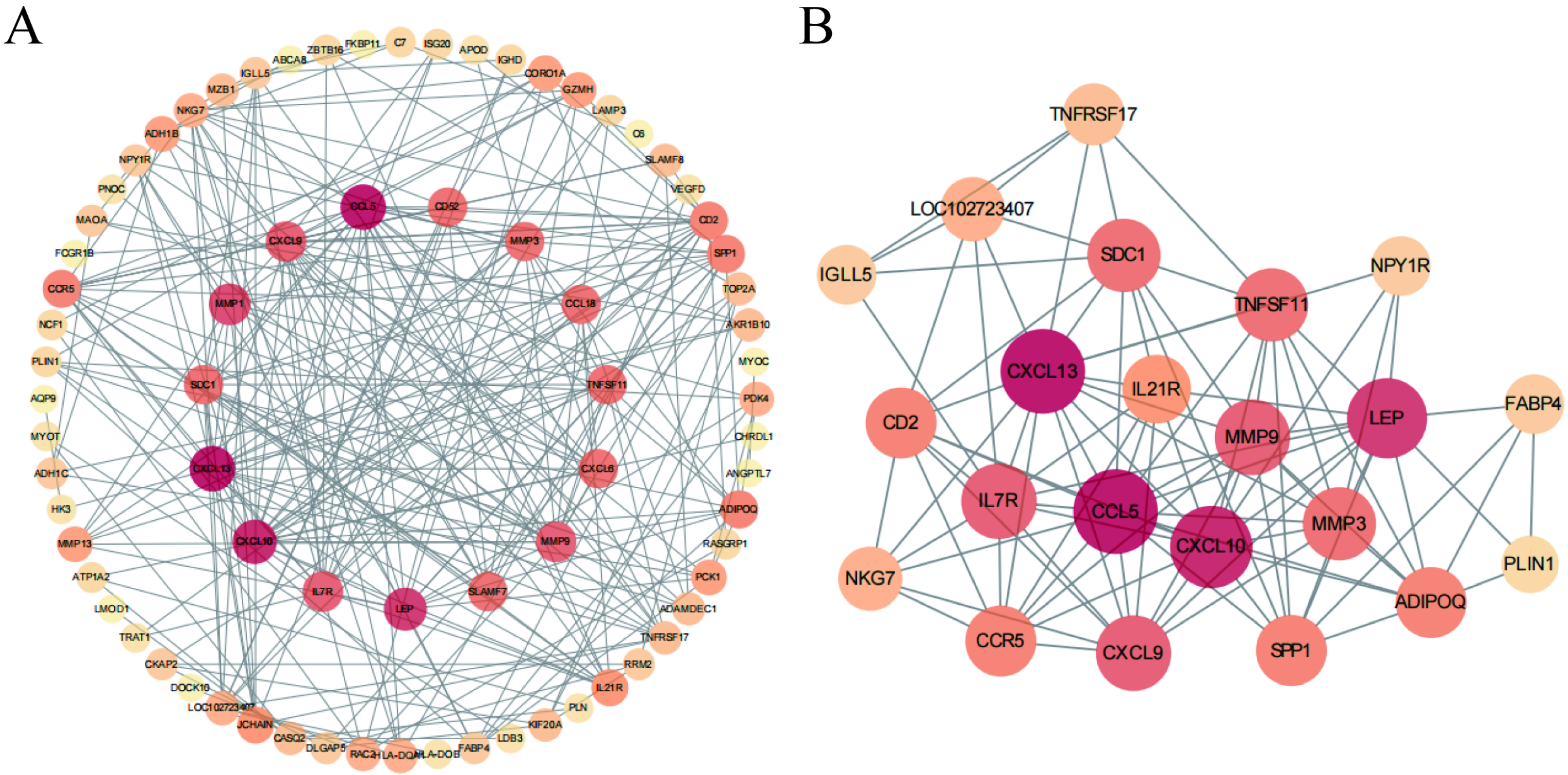

A
B
TNFRSF17
LOC102723407
IGLL5
SDC1
TNFSF11
NPY1R
CXCL13
IL21R
CD2
MMP9
LEP
FABP4
IL7R
CCL5
CXCL10
MMP3
PLIN1
NKG7
ADIPOQ
CCR5
CXCL9
SPP1
