# Supplementary figures and images for: Identification of SDC1 as a Key Regulator and Therapeutic Target in Rheumatoid Arthritis via JAK2‐STAT3 Pathway
Source: Int J Rheum Dis. 2026 Jan 29;29(2):e70524. doi: 10.1111/1756-185x.70524 (PMC12853146; doi:10.1111/1756-185x.70524)

GSE1919

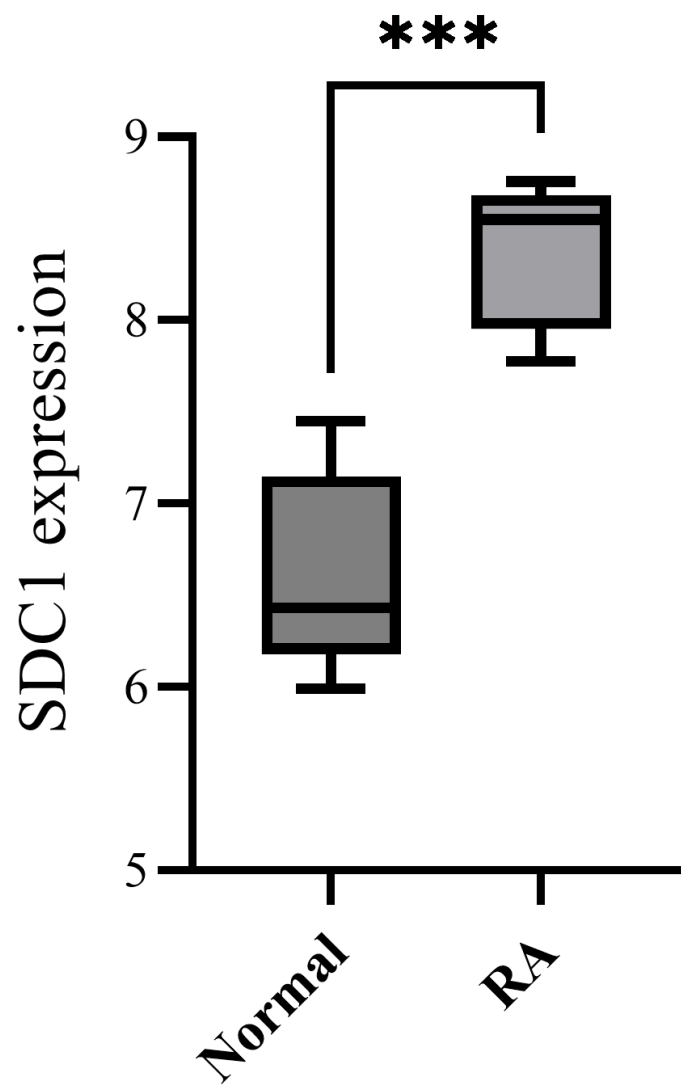

GSE12021

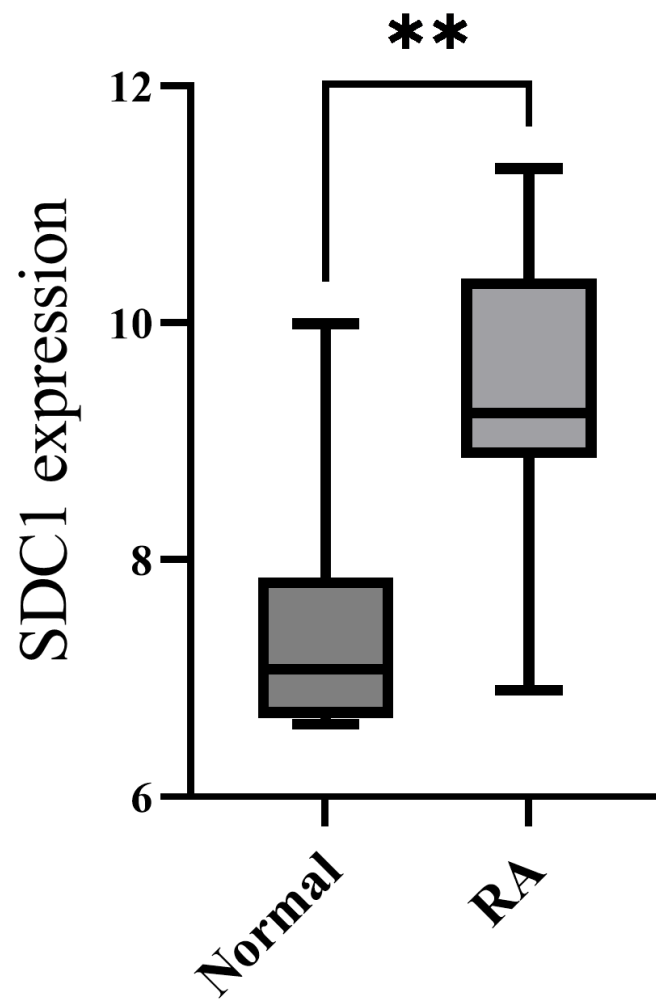

Supplement: Supplementary file 2 — Figure S2: Validation of Hub Genes SDC1. Comparison of SDC1 expression in normal and RA synovial tissues using two external datasets (GSE12021 and GSE1919). [file APL-29-e70524-s002.pdf]

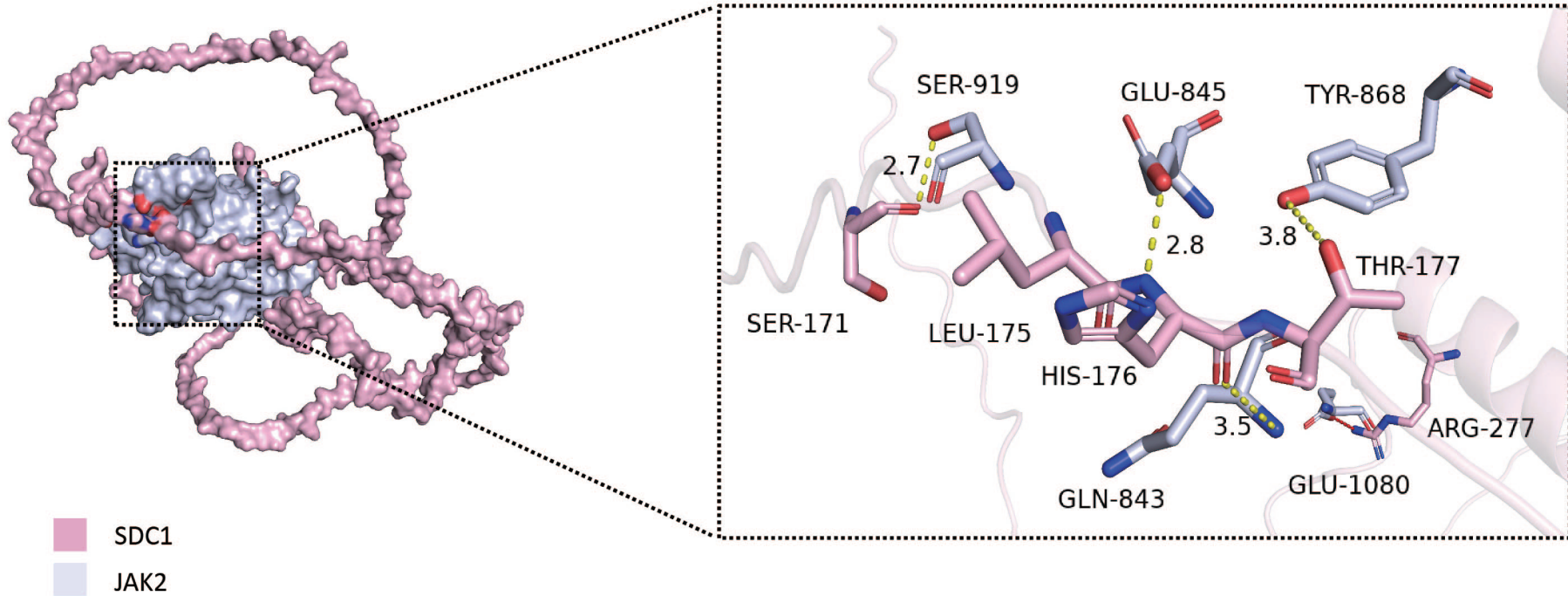

Supplement: Supplementary file 3 — Figure S3: The molecular docking results of SDC1 and JAK2. Predicted SDC1–JAK2 docking model highlighting key interface residues (SER‐171, SER‐919, GLU‐845 et al.) and the main stabilizing forces—hydrogen bonds and hydrophobic interactions. [file APL-29-e70524-s005.pdf]
