# Supplementary material for: Identification of SDC1 as a Key Regulator and Therapeutic Target in Rheumatoid Arthritis via JAK2‐STAT3 Pathway
Source: Int J Rheum Dis. 2026 Jan 29;29(2):e70524. doi: 10.1111/1756-185x.70524 (PMC12853146; doi:10.1111/1756-185x.70524)
Supplement: Supplementary file 5 — Table S2: Binding Energy of SDC1 and JAK family. [file APL-29-e70524-s001.docx]

Table 1 Binding Energy of SDC1 and JAK family

| Ligand: Protein | Receptor: Protein | Binding Energy |
| --- | --- | --- |
| JAK1 | SDC1 | -16.1 |
| JAK2 | SDC1 | -22.9 |
| JAK3 | SDC1 | -22.3 |
| TYK2 | SDC1 | -22.6 |
